# Coordination of prophage and global regulator leads to high enterotoxin production in staphylococcal food poisoning-associated lineage

**DOI:** 10.1128/spectrum.02927-23

**Published:** 2024-02-06

**Authors:** Yusuke Sato'o, Junzo Hisatsune, Fatkhanuddin Aziz, Nobuyuki Tatsukawa, Mari Shibata-Nakagawa, Hisaya K. Ono, Ikunori Naito, Katsuhiko Omoe, Motoyuki Sugai

**Affiliations:** 1 Department of Bacteriology, Hiroshima University Graduate School of Biomedical & Health Sciences, Hiroshima, Japan; 2 Antimicrobial Resistance Research Center, National Institute of Infectious Diseases (NIID), Tokyo, Japan; 3 Department of Veterinary Medicine, Faculty of Agriculture, Iwate University, Morioka city, Japan; 4 Laboratory of Zoonoses, Kitasato University School of Veterinary Medicine, Towada city, Japan; University of Calgary, Calgary, Canada

**Keywords:** *Staphylococcus aureus*, Staphylococcal enterotoxin, food poisoning, toxin production, Staphylococcal accessory regulator, bacteriophage

## Abstract

*Staphylococcus* species in food produce Staphylococcal enterotoxins (SEs) that cause Staphylococcal food poisoning (SFP). More than 20 SE types have been reported, among which Staphylococcal enterotoxin A (SEA) has been recognized as one of the most important SEs associated with SFP. However, the regulatory mechanisms underlying its production remain unclear. Previously, we identified a major SFP clone in Japan, CC81 subtype-1, which exhibits high SEA production. In this study, we attempted to identify the factors contributing to this phenomenon. Thus, we demonstrated that the attenuation of the activity of endogenous regulator, Staphylococcal accessory regulator S (SarS), and the lysogenization of a high SEA-producing phage contributed to this phenomenon in CC81 subtype-1. Furthermore, our results indicated that SarS could directly bind to the promoter upstream of the *sea* gene and suppress SEA expression; this low SarS repression activity was identified as one of the reasons for the high SEA production observed. Therefore, we revealed that both exogenous and endogenous factors may probably contribute to the high SEA production. Our results confirmed that SE production is a fundamental and critical factor in SFP and clarified the associated production mechanism while enhancing our understanding as to why a specific clone frequently causes SFP.

**IMPORTANCE:**

The importance of this study lies in its unveiling of a molecular regulatory mechanism associated with the most important food poisoning toxin and the evolution of Staphylococcal food poisoning (SFP)-associated clone. SFP is primarily caused by *Staphylococcus aureus*, with Staphylococcal enterotoxin A (SEA) being commonly involved in many cases. Thus, SEA has been recognized as a major toxin type. However, despite almost a century since its discovery, the complete mechanism of SEA production is as yet unknown. In this study, we analyzed an SEA-producing SFP clone isolated in East Asia and discovered that this strain, besides acquiring the high SEA-producing phage, exhibits remarkably high SEA production due to the low activity of SarS, an intrinsic regulatory factor. This is the first report documenting the evolution of the SFP clone through the coordinated action of exogenous mobile genetic factors and endogenous regulators on this notorious toxin.

## INTRODUCTION

Staphylococcal food poisoning (SFP) is a common foodborne disease worldwide. Basically, food poisoning can be classified under two types, namely, food intoxication and food infection, and SFP is classified under the former similar to foodborne botulism and cereulide-type *Bacillus cereus* food poisoning (1–3). The timeline of SFP occurrence is as follows (1, 4, 5): first, food is contaminated by Staphylococcal enterotoxin (SE) gene-positive staphylococci. Thereafter, as the bacteria grows, it produces SEs in the food. Next, humans consume the SE-contaminated food. This leads to the development of symptoms, including emesis and nausea, within a short period, usually 0.5–6 h. The sources of such bacterial contamination include humans, animals, and the environment. Furthermore, the frequently encountered causative foods include meat products, dairy products, and cereals. The main etiological pathogen of SFP is *Staphylococcus aureus*. However, cases of SFP caused by another emerging *Staphylococcus* species, namely, *S. argenteus*, have also been reported (6, 7).

In 1914, Baber reported that *S. aureus* can cause food poisoning, and in 1930, Dack et al. showed that food poisoning is caused by enterotoxin (1). Ever since Staphylococcal enterotoxin A (SEA) was first reported, more than 20 other types of SEs or SE-like toxins (SEls) have been reported (5, 8–10). The SE/SEl family represents a group of toxins that share a similar molecular structure (11). Among them, SEA is still given particular importance owing to its unique characteristics, the first of which is its toxicity. SEA shows stronger emetic activity against the *Suncus murinus* model than other classical SEs (12). It also shows stronger emetic activity against the primate model compared to recently characterized SEs (8, 13, 14). Second, the s*ea* gene shows sustainable expression in food (15, 16); our previous study also revealed that it is sufficiently produced in meat models (17). Third, it is associated with epidemiological data. Some studies involving food handlers and nasal cavities, which are common contamination sources, have shown relatively low SEA positivity rates (18–20). However, other studies have shown high positivity rates (>40%) for the *sea* gene in isolates from SFP outbreaks (19, 21–27). These positivity rates were often found to be higher than those of other SEs/SEls. Thus, SEA is not only frequently detected but also plays an important role in SFP cases. Particularly, it has been detected in three large SFP outbreaks (over 1,000 cases in the United States in 1985, approximately 4,000 cases in Brazil in 1998, and over 13,000 cases in Japan in 2000) (28–30). These epidemiological studies provide evidence that this toxin is the primary cause of Staphylococcal foodborne illnesses as well as occasional large-scale outbreaks. For these three reasons, SEA, which was first identified more than 90 years ago, is still recognized as the most important toxin associated with SFP.

SE/SEl genes are predominantly located on mobile genetic elements (MGEs) and immobilized former MGEs, such as prophages, *S. aureus* pathogenicity islands, enterotoxin gene clusters, and plasmids (5). Given that MGEs can move from once cell to another, horizontal MGE transfer is recognized as an important event in toxin production ability acquisition and bacterial pathogenicity. MGEs can also be transmitted across species borders, and this may contribute to SE production by *Staphylococcus* species other than *S. aureus*. Although most SE/SEl genes are present in MGEs, the expression of several SE genes is mainly controlled by the gene regulators encoded in the core genome of *Staphylococcus* (5, 31). These global regulators, including Staphylococcal accessory regulator (Sar) family members, two-component systems, and sigma factors, affect SE/SEl gene expression.

As described above, the regulatory mechanism is considered a unique factor that shapes SEA production characteristics; however, currently, it remains unclear. Previous studies have shown that there exist differences between the regulation of SEA and other SEs, indicating that SEA production is less endogenously regulated and more exogenously regulated by the bacteriophage (phage) effect (5). Specifically, SEA is located on a lysogenized phage (prophage), and its production amount differs according to the prophage type (1, 5). In other words, some phages enhance SEA production during their life cycle (5, 32). Additionally, higher SEA-producing prophages carry *sea* genes with two promoters, promoter 2 located within the phage late operon, and promoter 1, which is the original *sea* promoter, located close upstream of the *sea* gene. Even though promoter 1 is located in the phage genome, it is not related to the phage life cycle. In contrast, promoter 2 is associated with the phage life cycle and high SEA production. When DNA is damaged, the SOS response is initiated, resulting in changes in the life cycle of the phage from lysogenic to lytic. After entering the lytic cycle, the phage transcribes SEA mRNA together with its structural mRNA(s) in the late operon. This leads to enhanced SEA production in *S. aureus* lysogenized with a phage-carrying promoter 2, and reportedly, this phenomenon involves Recombinase A (RecA) and the SOS response (33). Previous studies have also suggested that the phage type has a major impact on SEA production (34). However, our previous studies have shown alternative possibilities as well as the existence of other unknown factors. In recent SFP outbreaks in Japan, CC81 subtype-1, which produces a large amount of SEA, was identified as the dominant lineage (19). Almost all isolates classified under this lineage carry the φSa3mw2 type prophage, which harbors *sea* promoter 2 (33). This suggests that SEA production is highly dependent on the presence of prophage. Conversely, we have also observed that CC81 subtype-1 isolates (No. 10, Nagasaki, and 01240) harboring φSa3mw2 type prophage produce larger amounts of SEA than MW2, a reference strain harboring the same φSa3mw2 type prophage (17). Therefore, based on these findings, in this study, we hypothesized that an additional hidden mechanism enhances SE production and, thereafter, attempted to elucidate this mechanism.

## RESULTS

### RecA affects SEA production but not all

**Fig 1:**
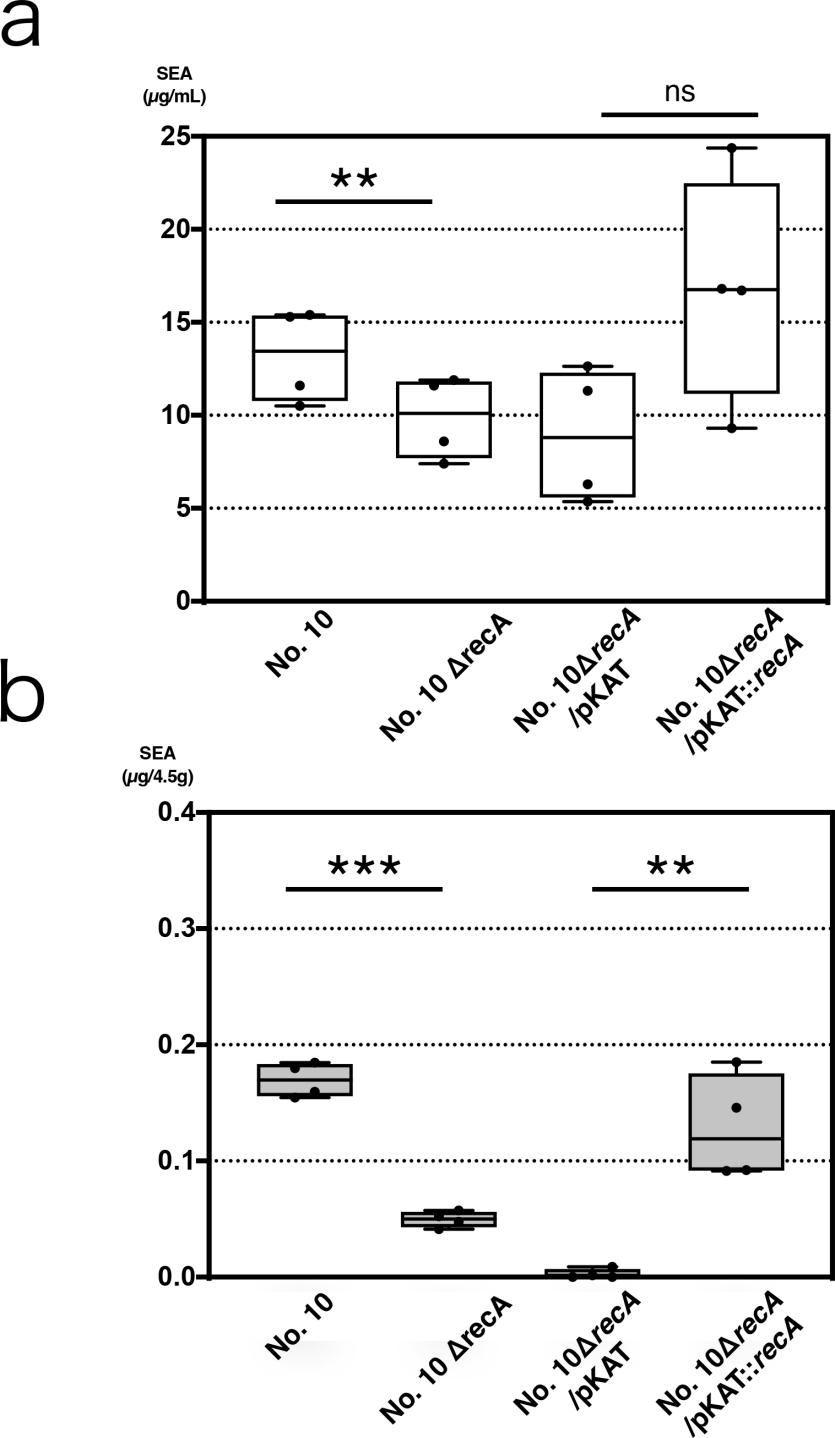
Effect of RecA on SEA production in No. 10. The SEA productivities of the wild-type strain and three *recA* mutants were quantified. Upper graph (1a), culturing in medium; lower graph (1b), culturing on meat; No. 10, Wild type; No. 10Δ*recA*, deletion mutant; No. 10Δ*recA*/pKAT, vector control; and No. 10Δ*recA*/pKAT::*recA*, complement mutant. All points from minimum to maximum are shown. Each culturing process was performed twice, and each culture was assayed via ELISA twice (*n* = 4/condition). In this study, SEA production in wild type No.10 was re-quantified. ns, not significant (*p* > 0.05); **p < 0.01; ***p < 0.001.

**Fig 2:**
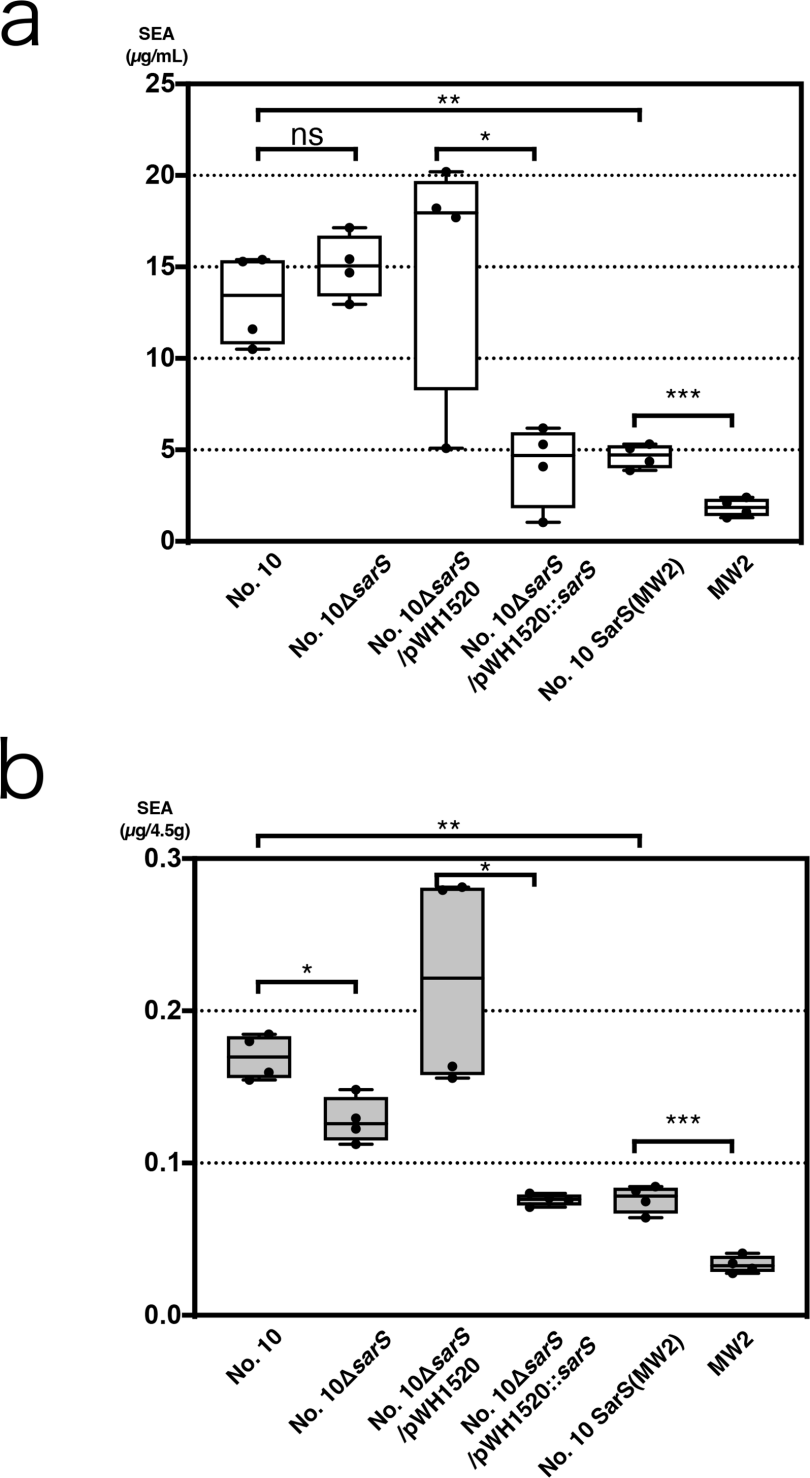
SarS effect on SEA production in No. 10. The SEA productivity of the wild type strain and four *sarS* mutants were quantified. Upper graph (2a), culturing in medium; lower graph (2b), culturing on meat; No. 10, wild type; No. 10Δ*sarS*, deletion mutant; No. 10Δ*sarS*/pWH1520, vector control; No. 10Δ*sarS*/pWH1520::*sarS*, complement mutant; No. 10 SarS (MW2), Allelic replacement mutant; and MW2, Wild type. The minimum to maximum and all other points are shown. Each culturing process was conducted twice, and each culture was assayed via ELISA twice (*n* = 4/condition). In this study, we re-quantified SEA production in wild-type No.10 and MW2. The values for wild-type No. 10 in this figure are same as shown in Fig. 1. ns, not significant (*p* > 0.05); *p < 0.05; **p < 0.01; ***p < 0.001.

Our previous study showed that CC81 sbutype-1 harbors a φSa3mw2 type prophage, which carries two *sea* promoters, namely, promoter 1 and promoter 2, and is integrated into the same φSa3 locus (19). Thus, we first examined the effect of phage on SEA production and the role of *sea* promoter 2 in this regard, using NaCl addition and RecA mutants. Reportedly, NaCl induces phage and increases *sea* expression (35). Accordingly, SEA production by CC81 subtype-1 with/without NaCl was assayed using enzyme-linked immunosorbent assay (ELISA). Fig. S1 shows that SEA production increased in the presence of NaCl in all four strains harboring the φSa3mw2 type phage. However, even in the presence of NaCl, three strains belonging to the CC81 subtype-1 (No. 10, Nagasaki, and 01240) showed more SEA production than MW2, a reference strain harboring φSa3mw2 that is classified as another CC, i.e., CC1. Next, we verified whether promoter 2 of *sea* and RecA, which is involved in SOS response, also influence SEA production in CC81 subtype-1 similar to that observed in the previous study (34). Thus, we constructed *recA* knockout, vector control, and *recA* complemented mutants from strain No. 10 and examined their growth curves. Thus, we observed that these mutants are similar to their parental strain (Fig. S2). We also assayed SEA productivity via ELISA. The results obtained are shown in Fig. 1 from which it is evident that *recA* deletion (No. 10 Δ*recA*) significantly decreased SEA production (*p* = 0.003) in the medium, while its complementation (No. 10ΔrecA/pKAT::*recA*) recovered the decrease in SEA production but not significantly (*p* = 0.09; Fig. 1A). This phenomenon was more pronounced in the meat model (Fig. 1B). Furthermore, significant differences were observed for this model for both the deficient (No. 10Δ*recA*) and complementary (No. 10ΔrecA/pKAT::*recA*) strains. However, SEA production by the *recA* deletion mutant in both the medium and meat models was still higher than that observed for wild-type MW2 (Fig. 2A and B, right lane). These findings suggested that phage and *sea* promoter 2 may partially influence SEA production, as previously described (34). However, the possibility that other factor(s) and promoter 1 may also influence SEA production, especially in this lineage, could not be ruled out.

### Staphylococcal accessary regulator S (SarS) plays an important role in high SEA production

Next, we compared the genome of No. 10 with that of MW2 to identify differences in the genetic background responsible for high SEA productivity. Thus, some sequence differences were observed. Specifically, the observed polymorphisms included the coding sequence of Protein A, SDR cell wall protein, alcohol dehydrogenases, and the toxin/anti-toxin system (*yoeB/yefM*), as was briefly mentioned in our previous study (36). Of these, we focused on the *sarS* (also known as *sarH1*) gene given that it is a global regulator and is reportedly associated with toxin production in *S. aureus* (37). Its nucleic acid and amino acid alignments are shown in Fig. S3 and S4, respectively. From these figures, it was evident that the C-terminal side of the SarS protein of No. 10, which is part of the helix-turn-helix DNA-binding domain, possesses 27 amino acid deletions. A single nonsense nucleotide polymorphism (c.670c > t, p.Q224*) was also identified in the second domain of SarS. Subsequently, we constructed an allelic replacement strain with MW2-type SarS and compared its SEA productivity with those of the wild-type, SarS deletion, SarS (MW2)-replaced, and complemented strains. While the vector control and complement strains showed relatively slow growth, the other strains showed growth patterns similar to that of the parental strain (Fig S2). Furthermore, our results indicated that the toxin productivity of *sarS* deletion No. 10 mutants (No. 10Δ*sarS*) was not significantly different from that of the parental strain in medium (Fig. 2A. *p* = 0.14). However, the SEA productivities of the vector SarS (MW2)-complemented mutant (No. 10ΔsarS/pWH1520::*sarS*) and mutant with chromosomally replaced MW2 type SarS (No. 10 SarS(MW2)) were significantly decreased in the medium compared with those of their counterparts (*p* = 0.04 and *p* = 0.006, respectively). Moreover, SEA productivity of the SarS allele-replaced mutant was higher than that of MW2 (*p* = 0.0003). A similar trend was observed in the meat model (Fig. 2B). Furthermore, complementation with SarS (MW2) and SarS (MW2) allelic replacement significantly reduced SEA production, compared to the SEA productivities of their counterparts (*p* = 0.01 and *p* = 0.002). However, the SarS deletion mutant showed significantly reduced SEA production in the meat model compared to the wild-type strain (*p* = 0.01), while no significant differences were observed in the medium. Additionally, the allelic replacement mutants showed higher SEA productivities than MW2 in this food model (*p* = 0.0005). These findings suggested that the SarS regulator is predominantly, but not entirely, responsible for the high toxin production observed in strain No. 10.

**Fig 3:**
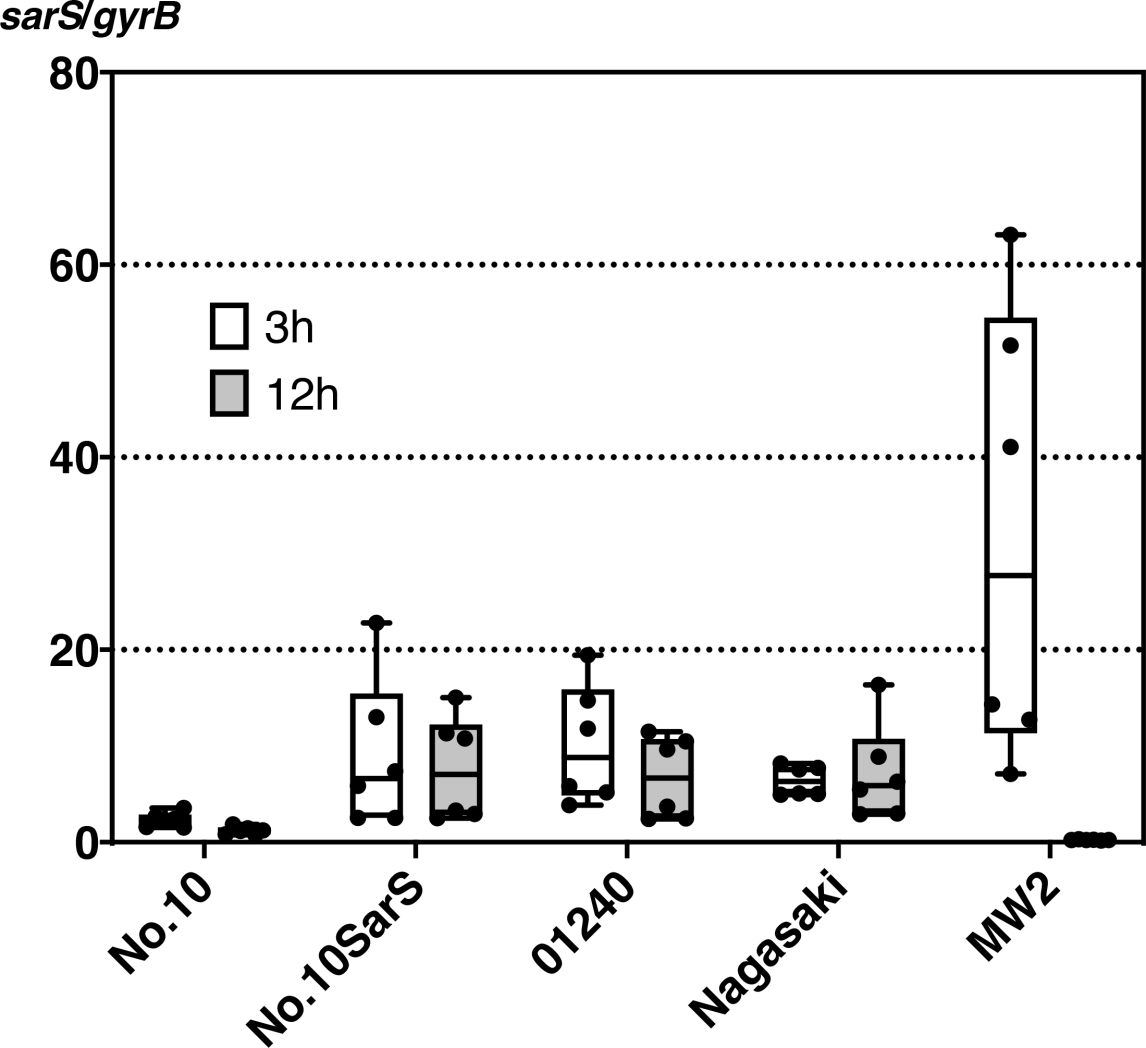
SarS expression levels of *S. aureus* strains used in this study. CC81 subtype-1 strains were compared with the MW2 strain. The *sarS* expression values normalized with *gyrB* are shown. White, 3 h (early-mid log phase); gray, 12 h (late log-stationary phase). No. 10 SarS, No. 10 SarS (MW2). The minimum to maximum and all other points are shown. All the culturing processes and all qPCR assays were performed two and three times, respectively (each sample, *n* = 6).

Next, we examined other strains to verify the universality of this phenomenon. Specifically, the *sarS* sequences of other 22 CC81 subtype-1 strains used in a previous study (19) were determined. Thus, no SarS gene truncation was observed except in No. 10, and the others had the same amino acid sequence as MW2. Even though our results showed that SarS was intact in these strains, previous results showing high toxin production (17) led us to consider the existence of another mechanism by which SarS influences SEA production. It is well known that SarS acts as an important activator for Staphylococcal protein A (Spa). Therefore, we performed western blot analysis for Spa and qPCR for SarS. Our groth curve analysis results indicated similar growth rates for all the examined strains (Fig. S2). Furthermore, as shown in Fig. S5, Spa production in the wild-type strain (No. 10) was lower than that in the chromosome allelic replacement mutant, No. 10 SarS (MW2), used in the above experiments (Lanes 1 and 2). The increase in Spa production following *sarS* allelic replacement indicated that the truncated SarS in strain No. 10 became partly dysfunctional. Furthermore, all the wild-type CC81 subtype-1 strains (lanes 1, 3, and 4) showed lower Spa expression than the non-CC81 strains (lanes 5 and 6). The results of qPCR (Fig. 3) also showed good agreement with the western blot data. SarS expression in the wild-type strain (No. 10) was lower than that in the allelic replacement strain [No. 10 SarS (MW2)]. Additionally, SarS mRNA was less abundant in CC81 subtype-1 strains than in MW2 strains in the early phase. In the case of the No. 10 strain, it harbors an abnormally low-activity protein, whereas strains 01240 and Nagasaki possess normal proteins. In both cases, SarS expression itself was reduced in the early log phase. This low expression level lasted for at least 12 h in a broth culture. In the case of MW2, SarS expression was significantly reduced after 12 h, suggesting that low SarS activity in the early growth phase of CC81 subtype-1 may be important for high SEA production in this group.

### SarS can bind the original promoter of the SEA gene

**Fig 4:**
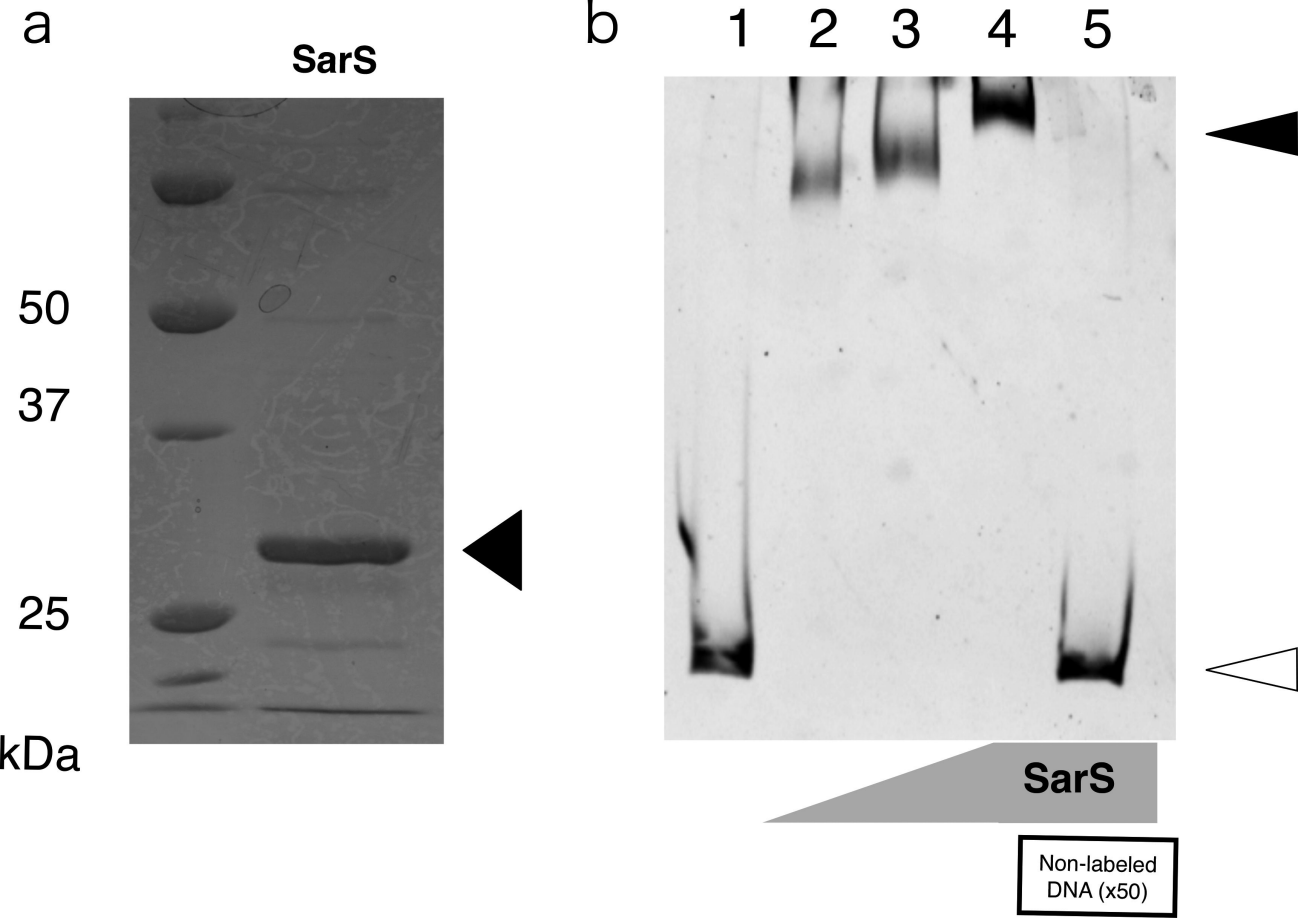
Gel shift assay of *sea* promoter DNA and rSarS protein. (**A**) Purified rSarS protein (Black arrowhead). The left lane represents the molecular marker (kDa). (**B**) Electronic mobility shift assay (EMSA) assay with the DNA probe and the purified protein. EMSA was performed using the *sea* promoter sequence DNA probe (50 ng/reaction) following incubation with various concentrations of rSarS (ng/reaction). The rSarS protein amounts in the reaction tubes were as follows: Lane 1, 0; lane 2, 1 ng; lane 3, 2 ng; and lanes 4 and 5, 10 ng. A 50-fold non-labeled DNA probe was added to lane 5 in the reaction sample. White arrowhead, non-bound DNA; black arrowhead, rSarS bound-DNA. The SEA promoter probe and rSarS protein were incubated in a medium containing 20 mM Tris, 50 mM NaCl, 1 mM EDTA, 1 mM dithiothreitol, 1 µg Poly (dI-dC), DTT, and pH, 7.5 (17). Electrophoresis was performed using 6% polyacrylamide gel and 0.5× TBE buffer at a constant voltage of 100 V for 2.5 h under cold conditions. Subsequently, the gel was imaged using the Molecular Imager FX system (Bio-Rad, Hercules, CA, USA). Representative image is shown.

Finally, we verified whether SarS protein could bind *sea* original promoter, promoter 1, and directly regulate SEA expression using a gel shift assay (electric mobility shift assay). Thus, we purified MW2 type recombinant SarS (rSarS) as shown in Fig. 4A. Subsequently, we reacted this protein with the cy3-labeled/non-labeled promoter 1 DNA fragment. The result obtained is shown in Fig. 4B. The binding of rSarS to the promoter sequence resulted in an upward band shift. However, this shift was negated in the presence of abundant non-labeled DNA. These findings suggested that rSarS (MW2) could directly bind to promoter 1 and repress SEA production.

## DISCUSSION

**Fig 5:**
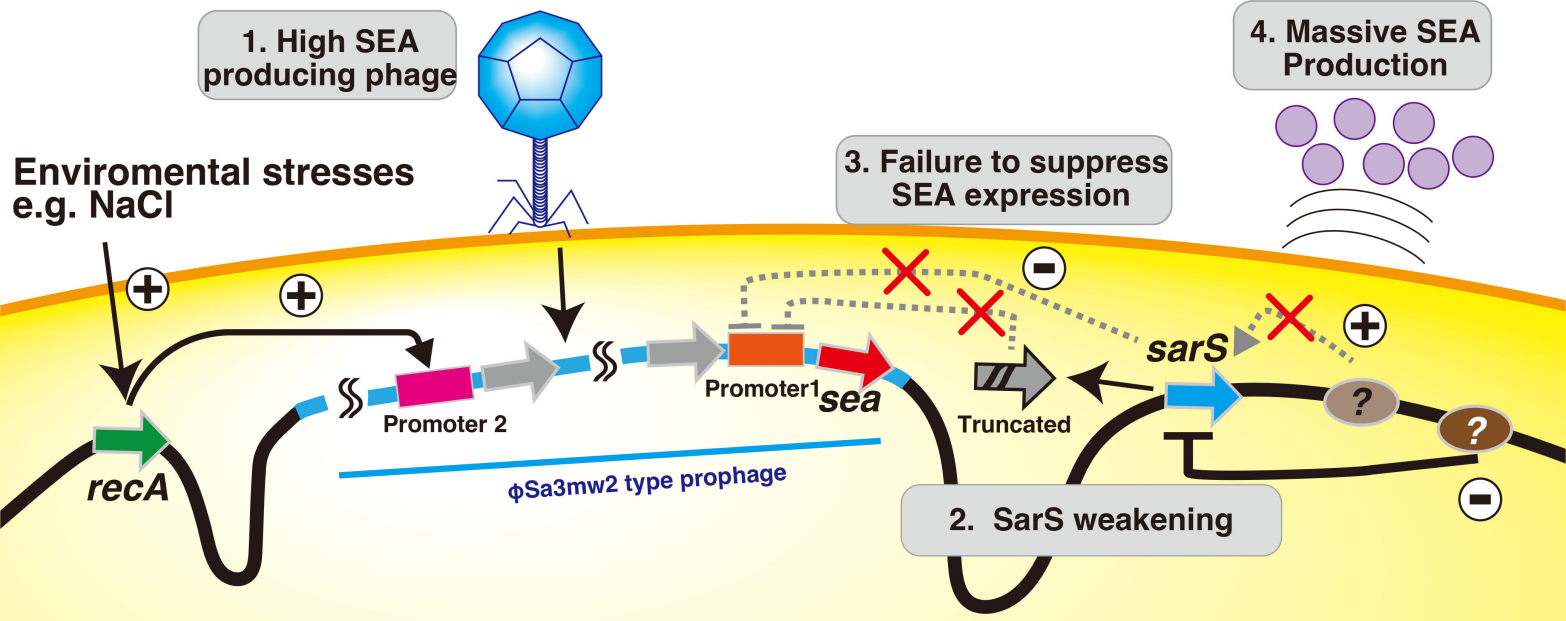
Schematic representation of the mechanism of high SEA production in CC81 subtype-1. The combined action of phage and SarS was found to be responsible for SEA production in CC81 subtype-1. The mechanism was as follows: 1. The high SEA production type phage, φSa3MW2, infected and lysogenized chromosome. This prophage was activated by stress, such that resulting from the addition of NaCl, which is one of the common factors in foods that leads to an increase in SEA production. 2. SarS was partly inactivated via truncation or repression. This happened in at least two ways, either owing to the presence of truncated SarS showing weak activity or low SarS expression, which possibly resulted from other unknown global regulator(s). 3. Normal SarS could bind promoter 1 and repress SEA production as shown in Fig. 4. The inhibitory effect was weakened because of the qualitative or quantitative decrease in activity. 4. The combination of the prophage (promoter 2) and the global regulator (promoter 1) led to excessive SEA production in this lineage. The common Siphoviridae shape, non-envelope, and icosahedral head and tail are shown. The blue region on the chromosome represents the prophage (φSa3mw2) locus. Even though we did not confirm the phage shape of φSa3MW2 in CC81 subtype-1 strains via electron microscopy, we predicted that the shape of this phage is similar to that of the Siphoviridae based on its genome size (approximately 40 kbp in CC81 subtype-1) (40)

Research progress in the field of SEs varies depending on the subfield. In the field of medicine, there has been significant advancement in the study of its superantigenic activity and molecular mechanisms (38). However, in the field of food hygiene, the research progress has been limited. Several studies have demonstrated efficient SEA production, and it has been suggested that this phenomenon can be attributed to phages (16, 34, 35, 39). However, it is still unclear whether it is universal for all SEA-producing strains or whether other factors may be involved in this process. In this study, we attempted to elucidate the factors that induce high SEA production in the dominant SFP clone in Japan, CC81 subtype-1. Based on our observations, the hypothetical schema shown in Fig. 5 was proposed. Specifically, we hypothesized that the low activity of SarS, accompanied by the involvement of the phage life cycle, is responsible for high SEA production. In other words, the coordination of a prophage and an endogenous regulator resulted in unusually high toxin production, at least in this SFP-associated clone.

Further, the regulation of virulence factors plays an important role in the pathogenicity of *S. aureus*, and it is well known that some SEs/SEls are controlled in a manner similar to the manner in which other toxin types are controlled. This toxin family, which includes TSST-1, is mainly regulated by Sar family proteins, two-component systems, and sigma factors (5, 41). However, unlike other SEs/SEls, SEA is exceptionally free from the control of most regulatory factors. A typical example is its independence with respect to the Agr-Rot system (42). Particularly, Agr is a two-component system in *S. aureus* consisting of RNAII and RNAIII, and a quorum sensing sensor. Once bacterial cell density reaches the threshold value, Agr alternates gene expression. Furthermore, Rot is a Sar family protein that binds to the promoter region of toxin genes and inhibits their transcription. At high cell densities, RNAIII transcription, which inhibits Rot translation, is initiated, resulting in Rot loss in the promoter region and the initiation of toxin gene transcription (5, 42). In contrast, surface proteins such as Spa show high expression during the early stages of growth, but their expression is subsequently suppressed with the upregulation of Agr (43). SEB, SEC, and SED are Agr-dependent toxins, whereas SEA, SEH, and SElJ are Agr-independent, among SEs/SEls (1, 5, 31, 44). The results of our previous study also showed that Rot has a reverse function and enhances SEH production at the transcriptional level (17). However, it has no effect on SEA production. Additionally, SEA is controlled by neither SaeR nor SigB (45). However, utilizing SrrAB, *S. aureus* is known to sense oxygen levels in its vicinity and regulate the expression of virulence genes (46). Even with SEs, the influence of oxygen has been reported (1), and toxin production is observed under both aerobic and anaerobic conditions although the optimal condition is aerobic. Therefore, the influence of SrrAB on SEA production is deemed considerable and cannot be overlooked. SEA production is rather affected by phage type and life cycle, and the principle is as follows (5, 32, 34): the SOS response mediated by RecA, which is triggered by DNA damage, activates dormant prophages on the host chromosome, leading to daughter phage production accompanied by toxin protein synthesis similar to Shiga toxin production in enterohemorrhagic *Escherichia coli* (47). This phage-associated phenomenon has been observed to a limited extent in some *sea*-carrying prophages (33, 34). Previous studies have also shown that the addition of NaCl, pH lowering, and the presence of mitomycin C induce *sea* gene expression (35, 39). In the present study, a similar trend of increased SEA production was observed following NaCl addition (Fig. S1; Fig. 5). However, a previous study showed that the addition of NaCl enhances mRNA expression but attenuates toxin protein amount (35). Reportedly, variations in SEA-producing strains affect the amount of SEA produced under stress conditions (16), and possibly, we observed a similar effect of strain differences in this study. This suggested that CC81 subtype-1 may possess the ability to produce more SEA under stress conditions. Additionally, the results of this study revealed that *recA* loss resulted in decreased SEA production (Fig. 1), suggesting that a similar mechanism associated with the phage cycle exists in this lineage and prophage. However, strain No. 10 with *recA* knockout still showed higher SEA production than MW2, which carries the same type of phage, indicating that strain No. 10 does not usually overexpress *RecA*, leading to a stronger SOS response. Induction via NaCl addition was also not considered to be more intense than induction owing to MW2 (Fig. S1). This is because MW2 increased induction by approximately twofold, whereas the remaining three CC81 subtype-1 strains showed increased induction to a lesser extent. In the experiments with the RecA mutant strains, there were two inconsistencies between the medium and food models (Fig. 1). First, the *recA* complement did not fully restore SEA production in the medium. Reportedly, in *Bacteroides*, plasmid complementation of *recA*-deficient strain does not completely restore phenotype (48). A similar phenomenon possibly occurred during our experiments in this study. Second, the effect of RecA was more significant for the food model. Stressors, such as a low pH and a high osmotic pressure, which are characteristic of foods, induce an SOS response. Furthermore, the absence of RecA, which plays a major role in stress response (49), may adversely affect growth or survival in food, resulting in a significant decrease in SEA production in the RecA-deficient strain.

As mentioned above, there are strong indications of the involvement of SrrAB based on existing research reports, yet the involvement of other global regulators in the mechanism of SEA production has not been reported to date. However, this study demonstrated that SarS inhibited SEA production. Therefore, this is the first report on the effect of coordination of global regulator and phage on SEA, which is an Agr-independent and quorum-sensing-independent toxin, whose production begins early during bacterial growth (50). Previous studies have shown that SarS expression in MW2 cells is high during the early culturing period and low during the late culturing period; this is consistent with the results of the present study (Fig. 3) (51). Furthermore, consistent with this result, Spa expression, which was observed during the early phase as mentioned above, was also high (Fig. 3; Fig. S5). A high level of SarS expression during the early culturing stage was expected to suppress SEA transcription and enhance Spa transcription, resulting in a lower level of SEA production and a higher level of Spa production during periods of low bacterial numbers, in the case of MW2. However, the CC81 subtype-1 showed low SarS expression during the early stages, suggesting that this lineage possibly produces more SEA and lower Spa during the early stage of bacterial proliferation. This may be advantageous for the early accumulation of toxins in food. Similar to *recA*, the *sarS* mutant strain also showed differences in SEA production between the food model and the culture medium. As shown in Fig. 2, the loss of truncated SarS did not affect SEA production in the medium but affected its production in food. This may also be attributed to stress response failure, such as RecA deficiency as mentioned above. Our previous study revealed a slight increase in SEH production with SarS deletion, suggesting that this mutant SarS is somewhat active (17). Moreover, one of the Sar family proteins, MgrA, is known to be involved in stress tolerance, and directly or indirectly interacts with other regulatory factors, including SarS, for stress response (49, 52, 53). Therefore, the SFP clone showed reduced SarS activity, possibly owing to its role in stress tolerance in food. However, this subject is beyond the scope of this study and requires further investigation in future studies. In addition, for strains within this lineage other than No. 10, we had only looked at the correlation between SarS and SEA production with Spa. Therefore, further research is needed to establish the causation of SarS and SEA production, including stress responses. However, this study at least demonstrates an inverse relationship between SarS and SEA production. It is estimated that the amount of SEA required for human vomiting is 100–200 ng (29), and the rapid production of SEA to amounts within this range is the most important issue in food poisoning outbreaks. This suggests that in food poisoning specifically, the CC81 subtype-1 clone evolves by suppressing SarS expression, but not completely, and the associated increase in SEA production results in rapid SEA accumulation in foods.

Both changes in the core genome and MGE acquisition are involved in *S. aureus* evolution (54). Regarding *S. aureus*-related infectious diseases, it is well known that some lineages with specific genetic backgrounds can spread across countries (55, 56). This study primarily showed that the same may be true for the genesis of SFP clones. In CC81 subtype-1, not only the acquisition of MGEs, including SEA and SEH, but also alterations in endogenous regulators, such as Rot and SarS, possibly led to its evolution as a more highly foodborne disease-associated group. Moreover, strains positive for both SEA and SEH have been isolated from food poisoning cases in South Korea (24), highlighting the possibility that closely related lineages may spread in East Asia. Emerging *S. aureus* infection clones have also been reported in recent years, probably owing to genomic changes (9, 57). Similarly, in SFP, there exists the possibility that new SFP clones originate from *S. aureus* and other staphylococci in the same manner. Ongoing molecular epidemiological surveillance will continue to be important to capture such genetic changes. Additionally, a deeper understanding of toxin production regulation and the survival strategies of toxigenic *S. aureus* in foods is also necessary.

## MATERIALS AND METHODS

### Bacterial strains and culture conditions

All the bacterial strains used in this study are listed in Table 1. In brief, *S. aureus* strains were cultured in brain heart infusion (BHI) broth, BHI agar, tryptic soy broth, and tryptic soy agar. *E. coli* strains were cultured in Luria-Bertani broth (1 L containing 10, 10, and 5 g of NaCl, trypticase peptone, and yeast extract, respectively). When necessary, ampicillin (final concentration, 100 µg/mL), chloramphenicol (final concentration, 10 µg/mL), tetracycline (final concentration, 5 µg/mL), D-xylose (final concentration, 1%, wt/vol), yeast extract (final concentration, 1%, wt/vol), and NaCl (final concentration, 3%) were added. All the materials were purchased from Becton Dickinson (Franklin Lakes, NJ, USA) or Wako Pure Chemical Industries (Osaka, Japan).

**TABLE 1:** Bacterial strains used in this study

*S. aureus*	Reference
No. 10	(58)
No. 10 Δ*sarS*	(17)
No. 10 Δ*sarS*/pWH1520	(17)
No. 10 Δ*sarS*/pWH1520::*sarS*	(17), SarS gene in vector was originated from another strain (59).
No. 10 SarS (MW2)	This study
No. 10 Δ*recA*	This study
No. 10 Δ*recA*/pKAT	This study
No. 10 Δ*recA*/pKAT::*recA*	This study
Nagasaki	(17, 19)
01240	(17, 19)
MW2	(60)
JP018 (previously named TF3033)	(61)
FDA S6	(62)
RN4220	(63)
*E. coli*	
DH5α	Takara
DH5α/pYS37	This study
BL21	Novagen
BL21 (DE3)	Novagen
BL21 (DE3)/pLysS/ pET24b::SarS	This study

### Growth curve

The growth curve method, which has been previously described (61), was used with some modifications. Specifically, the optical density of the culture medium (OD660) was measured using a spectrophotometer (SPECTRONIC 200; Thermo Scientific, Wilmington, DE, USA). BHI medium containing 1% yeast extract was used as the culture medium. After the inoculation of 1/100 vol of the overnight pre-culture media (40 µL) into 4 mL of fresh media in test tubes, all the test tubes were incubated at 37°C with constant agitation. Thereafter, temporal sampling (3 h, 6 h, and 12 h) was performed. The cultured media were diluted 5- to 10-fold with fresh media, and measurements were performed using SPECTRONIC 200.

### Conformation of mutation and domain analysis

A genomic comparison between No. 10 and MW2 was conducted in our previous study (36), and among them, SarS mutation was focused on in this study. We performed PCR and Sanger sequencing analyses using the primers listed in Table S1 to confirm the presence of a *sarS* nonsense mutation. The QuickTaq HS kit (Toyobo, Osaka, Japan) and Big-Dye terminator version 3.1 cycle sequencing kit (Applied Biosystems, Foster City, CA, USA) were used for PCR and sequencing, respectively. The CC81 subtype-1 strains used in our previous study were used for the SarS sequencing (19). Furthermore, we performed a domain search using InterPro software (64).

### Enzyme linked immune-solvent assay

ELISA samples from the medium (BHI with 1% yeast extract) and salted ham (meat model using a commercial meat product; Nippon Meat Packers, Inc., Osaka, Japan) were prepared as previously described (17) with some slight modifications to the meat model. Furthermore, approximately 4.5 g of salted ham was used as the meat model. After bacterial culturing, SEA on the meat samples was collected by washing with 1 mL of Dulbecco’s phosphate-buffered saline (pH 7.4) containing 0.1% (wt/vol) bovine serum albumin (Sigma, St. Louis, MO, USA). For pWH1520 and pWH1520::*sarS*, 1% xylose (wt/wt) was absorbed into the meat prior to bacterial culturing. The incubation lasted for 24 h, after which SEA ELISA, was performed as previously described (19).

### Genetic manipulation

We prepared several mutants of strain No. 10, as shown in Table 1. Notably, allelic replacement and gene complementation were performed as previously described (65). The plasmids and primers used in this study are listed in Table 2; Table S1. Furthermore, vector DNA for transformation was extracted from RN4220 using the manual alkaline SDS method or from BL21 using the FastGene Plasmid Mini Kit (Nippon Genetics Co., Ltd., Tokyo, Japan). Thereafter, electroporation was performed using the ELEPO21 system (NEPA GENE, Chiba, Japan) or BTX ECM830 system (BTX Harvard Apparatus, Inc., Holliston, MA, USA).

**TABLE 2:** Plasmids used in this study

Plasmids	Characteristics	Reference
pKFT	TS-vector	(65)
pKAT	Genetic manipulation	(66)
pGEMTeasy	Cloning vector	Promega
pYS37	pGEMTeasy::*sea* promoter, EMSA	This study
pYS4	pKFT::*recA* deletion fragment	This study
pYS27	pKFT::*sarS* (MW2 type)	This study
pYS28	pKAT::*recA*	This study
pET24b::SarS	SarS expression	This study

### RT-qPCR

BHI containing 1% yeast extract was used as the bacterial culture medium. After the inoculation of 1/100 vol of the overnight pre-culture media (100 µL) into 10 mL of fresh media in a test tube, bacterial culture was performed at 37°C with agitation. Temporary sampling (at 3h and 12 h) was performed. Thereafter, RNA extraction, reverse transcription, qPCR, and RT-qPCR quality check were performed as previously described (17). The primers used are listed in Table S1. The annealing temperatures for *gyrB* and *sarS* were 62 and 60°C, respectively.

### Protein A detection

Bacterial cultures and western blot analysis were performed as previously described (61). In brief, after the inoculation of 1/100 vol of the overnight pre-culture media (40 µL) into 4 mL of fresh media in a test tube, bacterial culture was performed for 24 h at 37°C with constant agitation. Thereafter, centrifugation (10,000 × *g*, 4°C, 5 min) was performed, and the supernatants were collected. SDS-PAGE was then performed using 10% gel. To block membranes, 5% skim milk (wt/vol, Morinaga Milk Industry Co., Ltd., Tokyo, Japan) in Dulbecco’s phosphate-buffered saline containing 0.05% (vol/vol) Tween 20 (PBST; Sigma-Aldrich, St Louis, MO, USA) was used. Furthermore, normal human IgG (final concentration, 2.5 µg/mL; LLC-Cappel Products, Irvine, CA, USA) and goat anti-human IgG (1/2,000 dilution, LLC-Cappel Products) were used as the primary and secondary antibodies, respectively. All washing and antibody reaction steps were performed using PBST as mentioned above.

### Electronic mobility shift assay

Recombinant SarS protein (rSasS) was prepared as previously described, with slight modifications (67). The materials used are listed in Tables 1 and 2. In brief, we used pET24b instead of pET14b and cloned *sarS* from JP018 (SRA accession number, DRR257760), which carries the same SarS as MW2. This vector was then transformed into BL21(DE3)/pLysS cells, and the recombinant protein was expressed using IPTG [isopropyl-β-d-thiogalactopyranoside (Wako, Osaka, Japan)]. Thereafter, the proteins were purified via His-tag purification using TALON Metal Affinity Resins (Takara, Otsu, Japan). We constructed a cy3 conjugated *sea* promoter DNA probe and performed an electrophoresis mobility shift assay (EMSA). The DNA probe was constructed via PCR and TA-cloning as described previously (17). The primers used are listed in Table S1. EMSA was also performed as previously described with some slight modifications (17). In brief, rSarS were incubated with the reaction buffer containing 20 mM Tris (final concentration), 50 mM NaCl, 5% glycerol, 1 mM EDTA, 1 mM dithiothreitol, and 1 µg Poly (dI-dC), and at pH 7.5. After incubation for 15 min without DNA at room temperature, labeled and unlabeled DNA was added to the reaction buffer, and the mixture was then incubated for 20 min at room temperature. Subsequently, the samples were subjected to electrophoresis performed on ice using 0.5× TBE and 5% native acrylamide gel.

### Statistical analysis

All statistical analyses were performed using GraphPad Prism 8 software (GraphPad Software Inc., San Diego, CA, USA). Paired *t*-test was performed to determine statistically significant differences between groups. *p* < 0.05 was considered statistically significant.

## SUPPLEMENTAL MATERIAL

Supplemental material spectrum.02927-23-s0001.pdf Figures S1 to S5 and Table S1.

ASM does not own the copyrights to Supplemental Material that may be linked to, or accessed through, an article. The authors have granted ASM a non-exclusive, world-wide license to publish the Supplemental Material files. Please contact the corresponding author directly for reuse.
