# Supplementary material for: Coordination of prophage and global regulator leads to high enterotoxin production in staphylococcal food poisoning-associated lineage
Source: Microbiol Spectr. 2024 Feb 6;12(3):e02927-23. doi: 10.1128/spectrum.02927-23 (PMC10913437; doi:10.1128/spectrum.02927-23)
Supplement: Supplemental material — Figures S1 to S5 and Table S1. [file spectrum.02927-23-s0001.pdf]

Figure S1

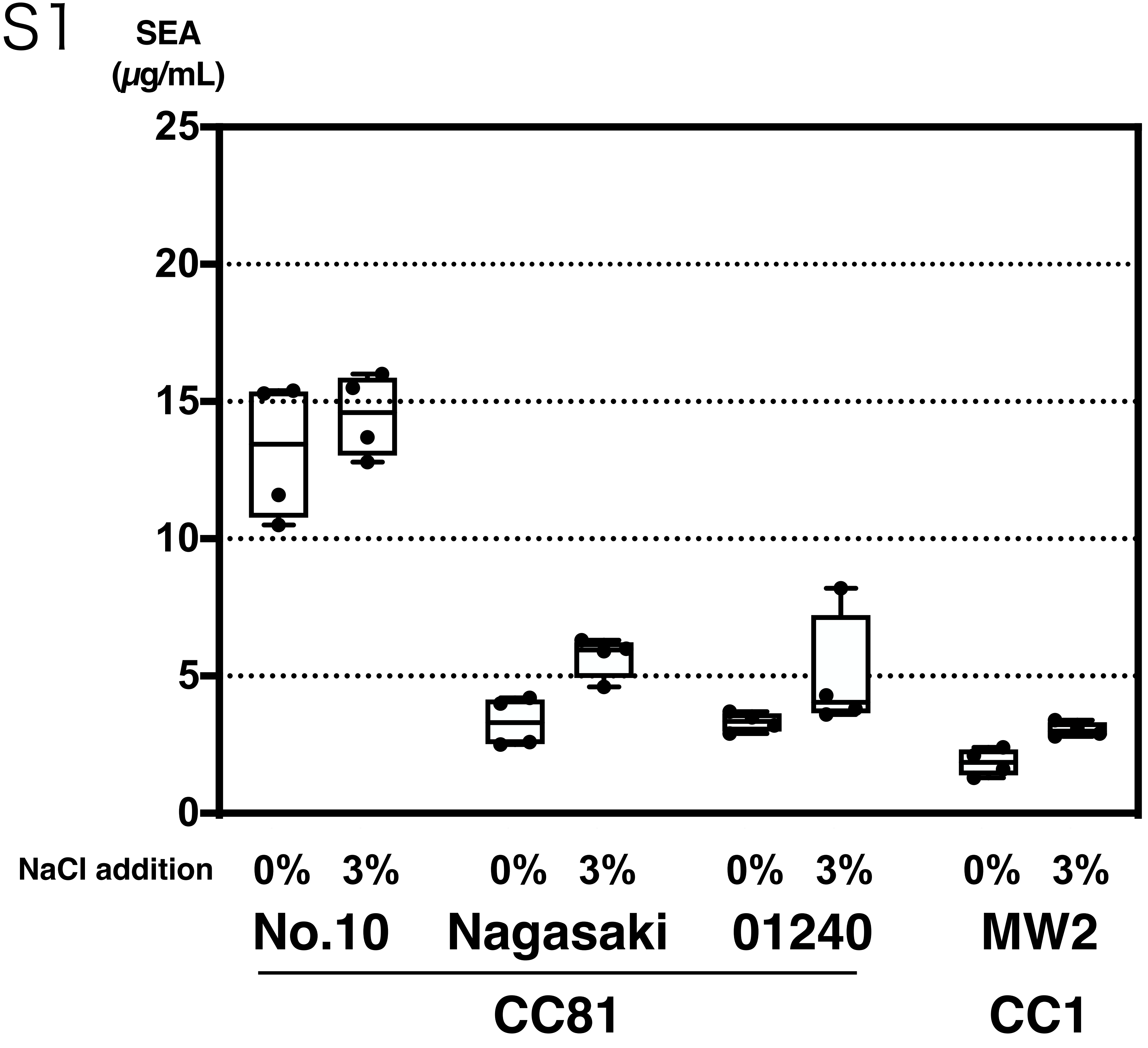

Supplemental Figure 1. NaCl effect on SEA production in *S. aureus* carrying ϕSa3mw2 type phage.The concentrations of SEA were examined in the broth model without the addition of NaCl and with the addition of 3% NaCl. Minimum to maximum and all points are shown. Each culture was conducted twice, and each culture was assayed by ELISA twice (n=4/each condition) . The Values in wild type No. 10 and MW2 without NaCl addition in this figure are same as those in Fig. 1

Figure S2

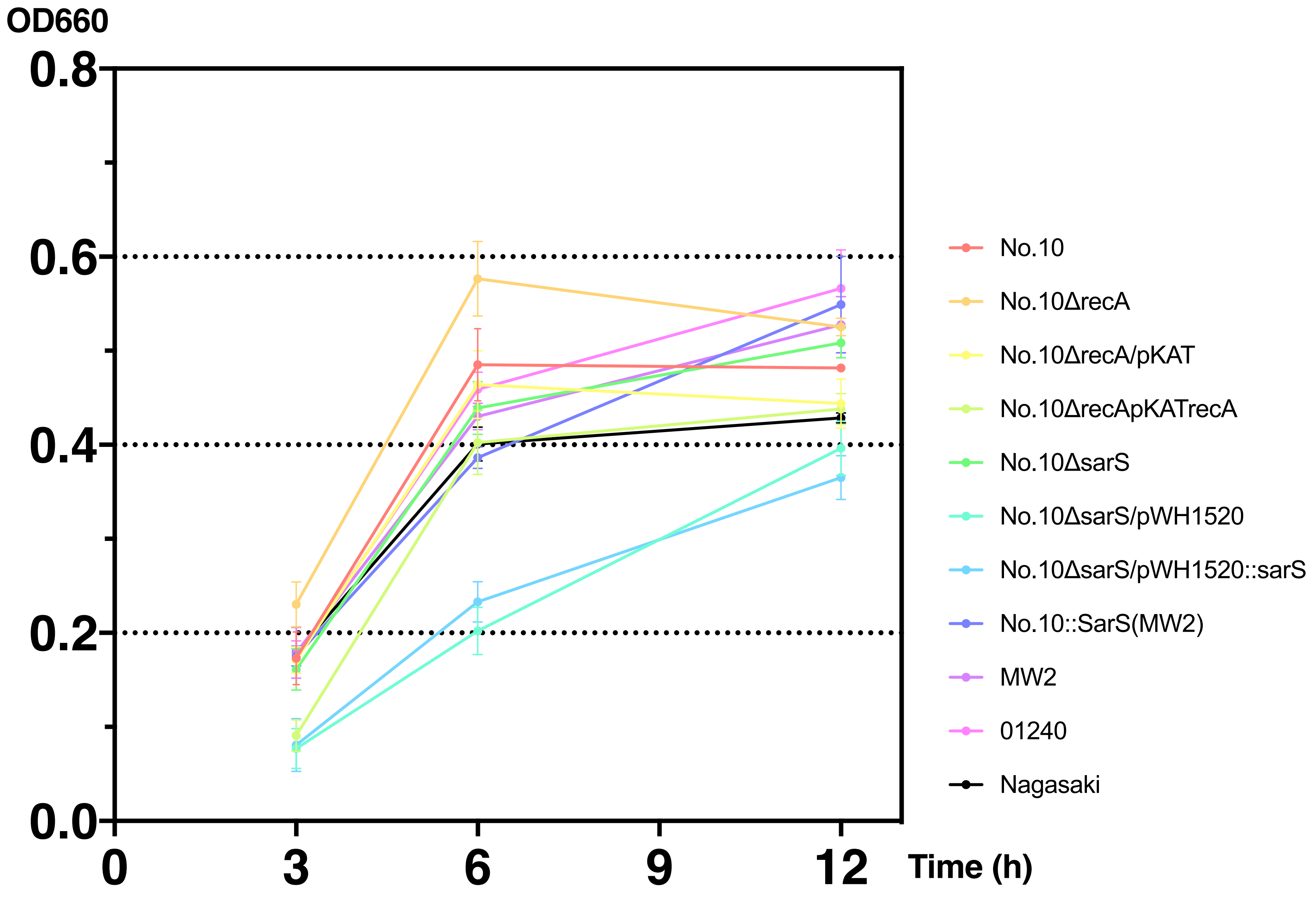

Supplemental Figure 2. Growth curve of *S. aureus* strains used in this study. Vertical line, Optical density (OD660); horizontal line, incubation time (3, 6, and 12hours). Three independent trials were performed (n=3). Average and standard error are shown.

Figure S3

```
SarS No.10 NA seq      1 ATGAAATATAATAACCATGACAAAATTAGAGATTTTATAATCATTGAAGCATATATGTTT     60
SarS MW2 NA seq        1 ATGAAATATAATAACCATGACAAAATTAGAGATTTTATAATCATTGAAGCATATATGTTT     60

SarS No.10 NA seq     61 CGTTTTAAGAAAAAAGTCAAGCCTGAAGTCGATATGACTATAAAAGAATTTATATTACTG    120
SarS MW2 NA seq       61 CGTTTTAAGAAAAAAGTCAAGCCTGAAGTCGATATGACTATAAAAGAATTTATATTACTG    120

SarS No.10 NA seq    121 ACTTATTTATTTCATCAGCAAGAAAACACACTTCCATTTAAGAAGATTGTTTCAGATTTA    180
SarS MW2 NA seq      121 ACTTATTTATTTCATCAGCAAGAAAACACACTTCCATTTAAGAAGATTGTTTCAGATTTA    180

SarS No.10 NA seq    181 TGTTATAAACAATCGGATTTAGTACAGCATATAAAAGTACTTGTGAAACATTCATATATT    240
SarS MW2 NA seq      181 TGTTATAAACAATCGGATTTAGTACAGCATATAAAAGTACTTGTGAAACATTCATATATT    240

SarS No.10 NA seq    241 AGTAAAGTTCGAAGTAAAATTGATGAGCGTAATACTTACATTTCAATATCTGAAGAACAA    300
SarS MW2 NA seq      241 AGTAAAGTTCGAAGTAAAATTGATGAGCGTAATACTTACATTTCAATATCTGAAGAACAA    300

SarS No.10 NA seq    301 CGAGAGAAAATTGCAGAACGTGTTACATTGTTTGATCAAATCATTAAACAATTTAACCTT    360
SarS MW2 NA seq      301 CGAGAGAAAATTGCAGAACGTGTTACATTGTTTGATCAAATCATTAAACAATTTAACCTT    360

SarS No.10 NA seq    361 GCAGATCAAAGTGAATCACAGATGATACCAAAAGATAGTAAAGAATTTCTAAACTTGATG    420
SarS MW2 NA seq      361 GCAGATCAAAGTGAATCACAGATGATACCAAAAGATAGTAAAGAATTTCTAAACTTGATG    420

SarS No.10 NA seq    421 ATGTATACAATGTATTTCAAGAATATTATCAAAAAACATCTAACATTAAGTTTTGTAGAA    480
SarS MW2 NA seq      421 ATGTATACAATGTATTTCAAGAATATTATCAAAAAACATCTAACATTAAGTTTTGTAGAA    480

SarS No.10 NA seq    481 TTCACAATTCTAGCTATTATCACTTCTCAAAATAAAAACATCGTTCTTCTTAAAGATTTA    540
SarS MW2 NA seq      481 TTCACAATTCTAGCTATTATCACTTCTCAAAATAAAAACATCGTTCTTCTTAAAGATTTA    540

SarS No.10 NA seq    541 ATTGAAACAATCCACCATAAATACCCTCAAACTGTTAGAGCTCTCAATAATTTAAAAAAG    600
SarS MW2 NA seq      541 ATTGAAACAATCCACCATAAATACCCTCAAACTGTTAGAGCTCTCAATAATTTAAAAAAG    600

SarS No.10 NA seq    601 CAAGGCTATCTAATAAAAGAACGCTCAACTGAAGATGAAAGAAAAATTTTAATTCATATG    660
SarS MW2 NA seq      601 CAAGGCTATCTAATAAAAGAACGCTCAACTGAAGATGAAAGAAAAATTTTAATTCATATG    660

SarS No.10 NA seq    661 GATGACGCGTAGCAAGACCATGCTGAACAATTATTAGCTCAAGTGAATCAATTATTAGCA    720
SarS MW2 NA seq      661 GATGACGCGCAGCAAGACCATGCTGAACAATTATTAGCTCAAGTGAATCAATTATTAGCA    720

SarS No.10 NA seq    721 GATAAAGATCATTTACATCTTGTTTTTGAATAA                               753
SarS MW2 NA seq      721 GATAAAGATCATTTACATCTTGTTTTTGAATAA                               753
```

Supplemental Figure3. Aliment of two SarS genes. One nonsense mutation (Red box, 670c>t) of *sarS* was found in No. 10, compared with that in MW2. Upper, SarS in No. 10; Downer, SarS in MW2. The full length from the start codon to the stop codon is shown.

Figure S4

1 **50**

MW2 MKYNNHDKIRDFIIIEAYMFRFKKKVKPEVDMTIKEFILLTYLFHQQENT

No. 10 MKYNNHDKIRDFIIIEAYMFRFKKKVKPEVDMTIKEFILLTYLFHQQENT

51 **100**

MW2 LPFKKIVSDLCYKQSDLVQHIKVLVKHSYISKVRSKIDERNTYISISEEQ

No. 10 LPFKKIVSDLCYKQSDLVQHIKVLVKHSYISKVRSKIDERNTYISISEEQ

101 **150**

MW2 REKIAERVTLFDQIIKQFNLADQSESQMIPKDSKEFLNLMMYTMYFKNII

No. 10 REKIAERVTLFDQIIKQFNLADQSESQMIPKDSKEFLNLMMYTMYFKNII

151 **200**

MW2 KKHLTLSFVEFTILAIITSQNKNIVLLKDLIETIHHKYPQTVRALNNLKK

No. 10 KKHLTLSFVEFTILAIITSQNKNIVLLKDLIETIHHKYPQTVRALNNLKK

201 **250**

MW2 QGYLIKERSTEDERKILIHMDDAQQDHAEQLLAQVNQLLADKDHLHLVFE

No. 10 QGYLIKERSTEDERKILIHMDDA*

Stop

Supplemental Figure4. Aliment of SarS protein. Only one nucleotide mutation of *sarS* was found in No. 10 (Sup. Fig. 2), compared with that in MW2. This nucleotide mutation lead to nonsense mutation in one of two domains in SarS protein. Upper, SarS in MW2; Downer, SarS in No.10. Under lines, predicted helix-turn-helix domain, which play a roll in DNA binding.

Figure S5

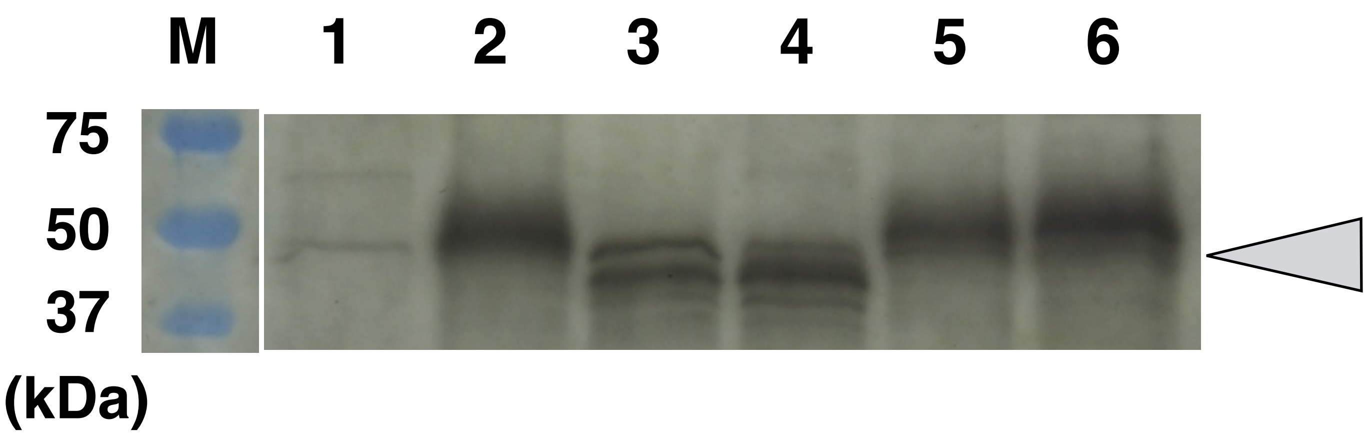

Supplemental Figure 5. Spa detection by using Western blotting. Arrowhead, Predicted molecular weight of SPA; M, Molecular maker (kDa); **1,** No.10**; 2,** No.10SarS(MW2)**; 3,** 01240; 4, Nagasaki**;** 5, MW2; 6, FDA 6S. Reproducibility has been confirmed, and representative result is shown.

Table S1. Primer used in this study

Primer name	Sequence (5'-3')	Characteristics
M13 primer M3	GTAAAACGACGGCCAGT	Construction of vectors
M13 primer RV	CAGGAAACAGCTATGAC	Construction of vectors
RecA1	CAGAGCTCCAGGAATGATGGT	Construction of mutants
RecA2	TCGAGCTCTTTCGACCATAT	Construction of mutants
RecA3	CCAATACTATACAAGGATTCA	Construction of mutants
RecA4	ATTTCATAAAGTCATATTGA	Construction of mutants
sarS1	GCCAAAGCTTATACATGGCTAGTCGG	Genetic manipulation and *sarS* sequence
sarS4	TCAAGGATCCATAGAAGGCGCTTTG	Genetic manipulation and *sarS* sequence
gyrB forward	AGGTCTTGGAGAAATGAATG	qPCR
gyrB reverse	CAAATGTTTGGTCCGCTT	qPCR
sarS forward	CCACCATAAATACCCTCAAACT	qPCR
sarS reverse	GTCTTGCTGCGCGTCAT	qPCR
T7 promoter-1	TAATACGACTCACTATAGGG	EMSA
T7 promoter-1 cy3	cy3-TAATACGACTCACTATAGGG	EMSA
SP6 promoter-1	CAAGCTATTTAGGTGACACTATAG	EMSA
SEA pro S	TTTGCTCACCTCTAAAGCATAATT	EMSA
SEA pro AS	GACAGGAAGCATACTGCAAGTGA	EMSA
